# Relationship between asthma, obesity and other comorbidities: the Australasian Severe Asthma Registry

**DOI:** 10.1186/s12931-026-03872-3

**Published:** 2026-08-21

**Authors:** Hayley A Scott, Joy Lee, Eve Denton, Adrian Barnett, Paul Griffiths, Peter G Middleton, Philip M Hansbro

**Affiliations:** 1 School of Biomedical Sciences and Pharmacy, The University of Newcastle, University Drive, Callaghan, NSW Australia; 2 Immune Health Research Program, Hunter Medical Research Institute, Level 2 West, Lot 1 Kookaburra Circuit, New Lambton Heights, NSW 2305 Australia; 3 Department of Respiratory and Sleep Medicine, Austin Health, Melbourne, VIC Australia; 4 School of Translational Medicine, Monash University, Melbourne, VIC Australia; 5 Asthma, Allergy and Clinical Immunology, Respiratory Medicine, Alfred Hospital, Melbourne, VIC Australia; 6 Department of Respiratory and Sleep Medicine, Mater Hospital, South Brisbane, QLD Australia; 7 Department of Respiratory Medicine, North Shore Hospital, Auckland, New Zealand; 8 Department of Respiratory and Sleep Medicine, Westmead Hospital, Sydney, New South Wales Australia; 9 Ludwig Engel Centre for Respiratory Research, Sydney, NSW Australia; 10 Centre for Inflammation, Faculty of Science, School of Life Sciences, Centenary Institute and University of Technology Sydney, Sydney, NSW Australia

**Keywords:** Asthma, Severe asthma, Comorbidity, Obesity, Asthma symptoms, Airway inflammation, Sex

## Abstract

**Background:**

The impact of obesity and sex on asthma outcomes and inflammation in the real-world setting is not completely understood. The aim of this study was to profile obesity and other comorbidities in a cohort of male and female adults with asthma; to understand the impact of obesity on asthma outcomes; and to examine sex-specific relationships between obesity and airway inflammation.

**Methods:**

We profiled comorbidities including obesity, as well as clinical asthma outcomes and airway inflammation, in patients with asthma enrolled in the Australasian Severe Asthma Registry. Data were analysed using *t*-tests (parametric data) or Wilcoxon rank sum tests (non-parametric data). Analysis of covariance (ANCOVA) was used to examine the effects of obesity and asthma severity on clinical and inflammatory outcomes.

**Results:**

We assessed 1,019 patients with severe (*n* = 882) and non-severe (*n* = 137) asthma. Patients with severe asthma were 67% more likely to have obesity, while patients with severe asthma and obesity were more likely to be female. Obesity was associated with a higher prevalence of asthma-related hospitalisation in the previous 12 months, in both severe (26.2% vs. 15.7%, *p* < 0.001) and non-severe asthma (12.5% vs. 3.1%, *p* < 0.001). Obesity was also associated with poorer asthma control and lung function, and higher sputum neutrophils. Interestingly, sputum %neutrophils correlated with body mass index in females (*r*_*s*_=0.370, *p* = 0.003), but not males (*r*_*s*_=0.092, *p* = 0.495). Neutrophilic airway inflammation was associated with poorer lung function and asthma control.

**Conclusions:**

This study demonstrates that obesity is associated with a significantly worse clinical asthma presentation in the real-world setting and increases the risk of asthma-related hospitalisation. Critically, our findings suggest that obesity is associated with increased neutrophilic airway inflammation in females, but not males, providing some insight into possible sex-related differences in severe asthma.

**Supplementary Information:**

The online version contains supplementary material available at https://doi.org/10.1186/s12931-026-03872-3.

## Background

Asthma affects approximately 300 million people worldwide, with 5–10% of people with asthma living with severe asthma [1–4]. Severe asthma significantly increases asthma-related burden [5] and accounts for 60% of asthma-related healthcare expenditure [6], corresponding to a 10-fold higher cost per patient compared with those with non-severe asthma.

Obesity prevalence has increased rapidly since the 1980’s, now affecting 1 in 8 people globally [7]. Obesity increases the risk of a wide range of comorbidities, including asthma [8]. In addition to increasing asthma risk, there is some evidence that obesity may also contribute to worse asthma outcomes. Indeed, obesity is a leading risk factor for disability adjusted life years in people with asthma and is estimated to account for almost half of asthma-related deaths globally [9]. Because of the substantial negative impact obesity has on respiratory morbidity and mortality, it is critical to better understand the influence of obesity on asthma outcomes in the real-world setting, as well as the mechanisms through which this occurs, in order to optimise clinical care and health outcomes.

The mechanisms involved in the association between obesity and asthma are not fully understood, however there is evidence that both obesity and a high fat diet (which is more common in people with obesity) independently induce inflammatory changes that underpin asthma pathogenesis [10–12]. Other proposed mechanisms include the presence of comorbidities including metabolic syndrome, female sex hormones, gastro-oesophageal reflux disease (GORD) and obstructive sleep apnoea (OSA), as well as mechanical effects of excess weight on lung physiology and the microbiome [13, 14]. It is likely a combination of factors that are responsible, however these relationships are complex and poorly understood.

Here, we interrogate the Australasian Severe Asthma Registry (ASAR) to profile obesity and other co-morbidities in this large real-world cohort of adults from Australia and New Zealand with severe and non-severe asthma (> 1,000), to better understand the clinical implications of comorbid asthma and obesity within the real-world setting and explore whether airway inflammation and sex influences asthma outcomes. We hypothesised there would be a higher prevalence of obesity and other comorbidities in patients with severe asthma. We also hypothesised that people living with both severe asthma and obesity would be more likely to be female, have poorer clinical asthma outcomes, elevated airway inflammation, and a higher prevalence of other comorbidities, compared with people living with severe asthma but without obesity.

## Methods

Data were obtained from the ASAR, a clinical quality registry that encompasses both cross-sectional and prospective longitudinal data collected from adults with severe and non-severe asthma across 28 sites in Australia and New Zealand. Prior to 2019, all patients provided written, informed consent. To ensure a representative population, a transition to opt-out consent was approved by the Australian Commission for Safety and Quality in Health Care in 2019. ASAR is under the governance of the Thoracic Society of Australia and New Zealand (TSANZ). Recruitment commenced in 2013, with systematic, de-identified, annual data collection via an electronic data capture system (REDCap) [15]. Ethical approval was obtained according to each site’s Human Research Ethics Committee in Australia (2019/ETH03836) and New Zealand (2/CEN/69/AM09).

Patients were *≥* 18 years old with confirmed asthma demonstrated by variable airflow obstruction (VAO), either at their first visit or documented within the past 10 years. Patients with severe asthma were on the highest level of recommended treatment [maximal inhaled corticosteroid (ICS) therapy with second controller] and had poor asthma control, as described previously [16]. The comparison group was adults with non-severe asthma who were also prescribed maintenance inhaled controller therapy and had partially-to-well controlled asthma (Asthma Control Questionnaire, ACQ6 < 1.5 [17] or Asthma Control Test, ACT > 20) [18], and stable disease with no infection, exacerbation or change in preventer therapy in the previous 4 weeks. Additional detail is outlined in the Supplementary Methods.

### Registry assessments

Data collection included demographics, body mass index (BMI), asthma and allergy history, smoking history, comorbidities, respiratory medications, exacerbation history and health care resource utilisation. Height and weight were measured without shoes. The asthma-related quality of life questionnaire (AQLQ) [17] and ACQ6 [19] were administered. Asthma exacerbations in the previous year were quantified as previously defined [20]. Clinical assessments included spirometry, fractional exhaled nitric oxide (FeNO) and induced sputum (if available). Induced sputum was collected using 4.5% hypertonic saline and processed according to Gibson [21]. Eosinophilic asthma was defined as sputum %eosinophils ≥ 3% and/or FeNO ≥ 30ppb. Neutrophilic asthma was defined using age-adjusted sputum neutrophil cut-points according to Brooks [22]. 

### Assessment of comorbidities

Diagnosis of comorbidities was by physician assessment, including:

- Pulmonary and upper airways: allergic rhinitis, chronic rhinosinusitis, nasal polyps, vocal cord dysfunction, OSA, bronchiectasis, chronic obstructive pulmonary disease (COPD), hyperventilation dysfunctional breathing, allergic bronchopulmonary aspergillosis; and.
- Extra-pulmonary: Obesity, diabetes, cardiovascular disease, anxiety, depression, GORD, osteoporosis, eczema and anaphylaxis.

### Statistical analysis

Data were analysed using Intercooled Stata 18.5 (StataCorp) with a significance threshold of 0.05. Demographic characteristics were summarised as percentages for categorical variables, mean (standard deviation) for parametric variables, or median (interquartile range) for non-parametric variables. Patients with severe *versus* non-severe asthma were compared with a *t*-test (parametric data) or Wilcoxon rank sum test (non-parametric data). Group comparisons of proportions were compared using Pearson’s Chi-squared test. Correlations were examined using the Spearman rank test (r_s_).

The effects of obesity and asthma severity on clinical and inflammatory outcomes were analysed with analysis of covariance (ANCOVA). The robust variance estimator was applied to account for outcomes being skewed. Models were adjusted for asthma severity (severe/non-severe), obesity (yes/no), inhaled corticosteroid dose (beclomethasone dipropionate equivalents/day), oral corticosteroid dose (mg/day), biologic use (yes/no) and smoking status (never/ex/current), except in models where that particular variable was the dependent variable. Results for group comparisons were presented as mean differences with 95% confidence intervals. An interaction between asthma severity and obesity was completed to test whether clinical asthma outcomes and inflammation differed according to these variables; this model was also adjusted for the same variables outlined above.

Change in asthma outcomes from baseline to 12 months was examined in patients with severe asthma, grouped according to biologic medication use and obesity status. Statistical analysis was conducted using ANOVA with post-hoc *t*-test, or Kruskal-Wallis with post-hoc Wilcoxon rank sum test. To account for multiple comparisons in the longitudinal data, *p*-values < 0.017 were considered statistically significant.

## Results

### Patient characteristics

Patients were enrolled into ASAR between 2013 and 2023. After excluding 55 patients who did not have baseline data available for height, weight and/or asthma severity, there were 882 patients with severe asthma and 137 with non-severe asthma (Table 1). Clinical characteristics of the severe and non-severe asthma cohorts were similar. Mean age at enrolment was 54 years and mean age at asthma diagnosis was 23 years.

**Table 1:** Patient characteristics, according to asthma severity

Characteristic	Severe asthma (*n* = 882)	Non-severe asthma (*n* = 137)	*P*-value
BMI (kg/m^2^) (*n* = 1,019)	30.9 (8.1)	28.3 (5.7)	**< 0.001**
Underweight, n (%)	16 (1.8%)	1 (0.7%)	**< 0.001**
Normal weight, n (%)	182 (20.6%)	37 (27.0%)	
Overweight, n (%)	261 (29.6%)	59 (43.1%)	
Obese Class I, n (%)	213 (24.2%)	22 (16.1%)	
Obese Class II, n (%)	105 (11.9%)	12 (8.8%)	
Obese Class III, n (%)	105 (11.9%)	6 (4.4%)	
Sex (n, %female) (*n* = 1,017)	551/880 (62.6%)	79/137 (57.7%)	0.267
Age (years) (*n* = 1000)	54.7 (14.8)	54.1 (16.6)	0.677
Age at asthma onset (years) (*n* = 881)	22.9 (19.2)	22.9 (20.3)	0.969
Ethnicity (*n* = 890)			**< 0.001**
Caucasian	653/761 (85.8%)	99/129 (76.7%)	
Aboriginal	7/761 (0.9%)	1/129 (0.8%)	
Pacific Islander	8/761 (1.1%)	1/129 (0.8%)	
Asian	56/761 (7.4%)	26/129 (20.2%)	
Southeast Asian	4/761 (0.5%)	0/129 (0%)	
Other	33/761 (4.3%)	2/129 (1.6%)	
Respiratory Medications			
ICS dose (BDP µg/day) (*n* = 1,018)	1600 (1000, 2000)	800 (400, 1000)	**< 0.001**
Daily OCS use	229/860 (26.6%)	5/133 (3.7%)	**< 0.001**
OCS dose for OCS users (mg/day) (*n* = 222)	10 (5, 13)	10 (8, 25)	0.572
Anti-IgE (Omalizumab)	110/849 (13.0%)	0/131 (0%)	**< 0.001**
Anti-IL-5 (Mepolizumab/Benralizumab)	200/870 (23.0%)	0/131 (0%)	**< 0.001**
Anti-IL-4 (Dupilumab)	19/854 (2.2%)	0/131 (0%)	0.085
Smoking status (n, %)			
Never smoked	519/854 (60.8%)	85/134 (63.4%)	0.228
Ex-smoker	305/854 (35.7%)	48/134 (35.8%)	
Current smoker	30/854 (3.5%)	1/134 (0.8%)	
Pack years of current and ex-smokers (*n* = 372)	11 (4, 27)	7 (1, 17)	**0.009**

*Definition of abbreviations*: *BDP*  Beclomethasone dipropionate, *BMI*  Body mass index, *ICS*  Inhaled corticosteroid, *OCS*  Oral corticosteroid. Underweight = BMI < 18.5 kg/m^2^; Normal weight = BMI 18.5–24.9 kg/m^2^; Overweight = BMI 25–29.9 kg/m^2^; Obese Class I = BMI 30–34.9 kg/m^2^; Obese Class II = BMI 35–39.9 kg/m^2^; Obese Class III = BMI ≥ 40 kg/m^2^

Patients with severe asthma had a higher BMI (30.9 ± 8.1 kg/m^2^) compared with those with non-severe asthma (28.3 ± 5.7 kg/m^2^, *p* < 0.001) (Table 1) and were 1.64 times more likely to have obesity. Patients with severe asthma had a higher daily dose of ICS, more prevalent use of daily oral corticosteroid and biologic (anti-IgE, anti-IL-5) medication, more severe airflow limitation, worse asthma control, more frequent asthma exacerbations, worse asthma-related quality of life, and higher sputum %eosinophils (Table 1). Neutrophilic asthma [22] was also more prevalent in severe asthma than in non-severe asthma (19.3% vs. 3.6%, *p* = 0.045) (Table S1).

Patients with severe asthma had a higher recorded prevalence of pulmonary and upper airway comorbidities, including nasal polyps (19.2% vs. 10.2%, *p* = 0.001), OSA (15.8% vs. 7.3%, *p* = 0.009), bronchiectasis (7.7% vs. 2.9%, *p* = 0.042) and COPD (9.4% vs. 3.7%, *p* = 0.026) (Table S1). This group also had a higher prevalence of GORD (42.6% vs. 32.9%, *p* = 0.030) (Table S1).

### Impact of obesity and asthma severity on clinical asthma outcomes

**Fig. 1:**
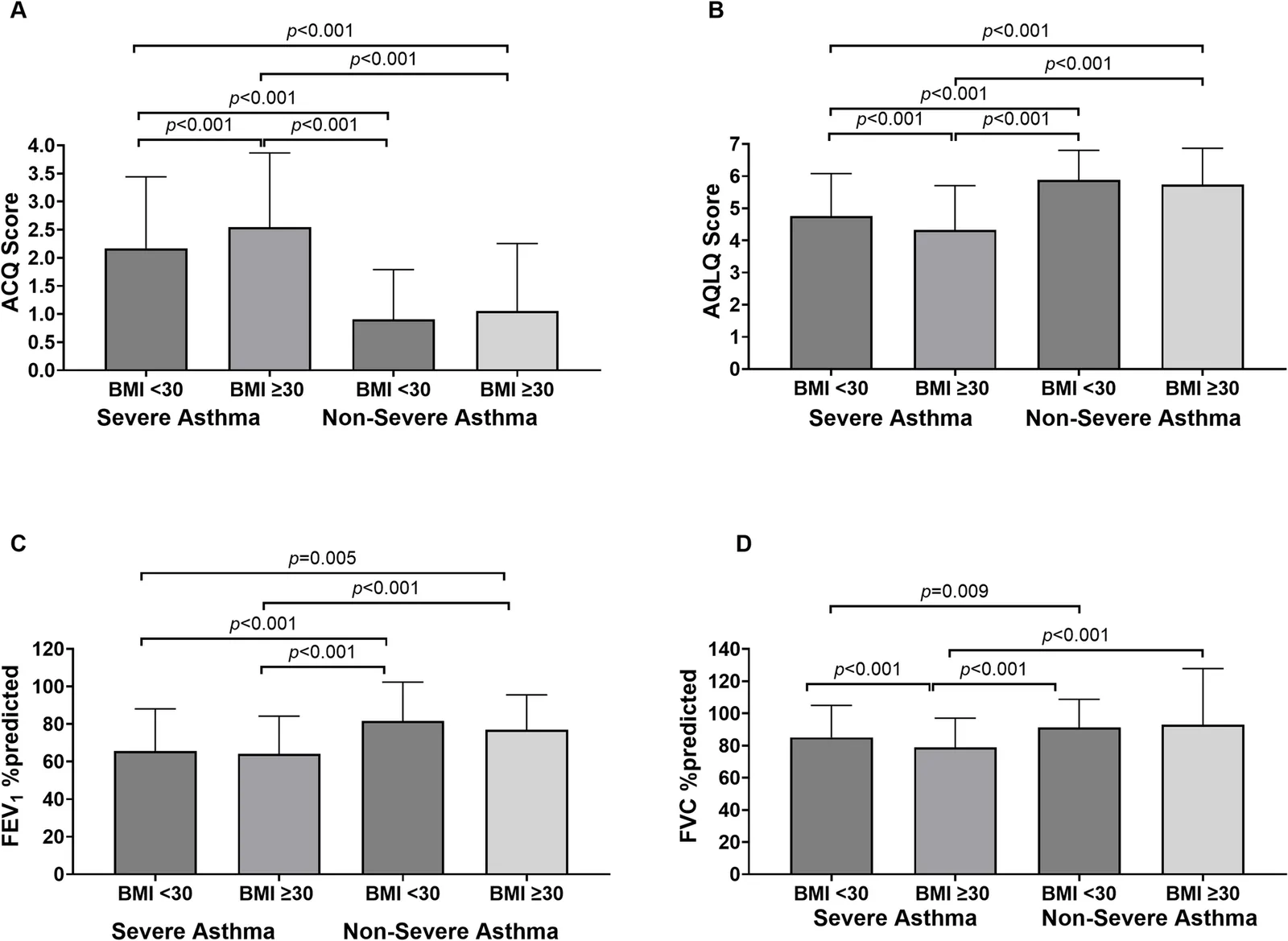
Mean (SD) (**a**) asthma control questionnaire (ACQ) score, (**b**) asthma-related quality of life (AQLQ) score, (**c**) pre-bronchodilator %predicted forced expiratory volume in 1 s (FEV_1_), and (**d**) pre-bronchodilator %predicted forced vital capacity (FVC), according to asthma severity and body mass index (BMI)

Asthma was more poorly controlled (ACQ) in patients with *versus* without obesity [mean difference, MD (95% CI) = 0.33 (0.16 to 0.50), *p* < 0.001] and in patients with severe asthma *versus* non-severe asthma [MD (95% CI) = 1.21 (0.99 to 1.43), *p* < 0.001; Fig. 1a; Table 2, Table S4]. Asthma-related quality-of-life (AQLQ) was also worse in patients with *versus* without obesity [MD (95% CI) = -0.36 (-0.54 to -0.19), *p* < 0.001] and in patients with severe asthma *versus* non-severe asthma [MD (95% CI) = -1.04 (-0.44 to -0.19), *p* < 0.001] (Fig. 1b; Table 2, Table S4), and this pattern was consistent across all AQLQ domains. Higher BMI correlated with worse ACQ (*r*_*s*_=0.196, *p* < 0.001) and worse AQLQ (*r*_*s*_=-0.196, *p* < 0.001).

**Table 2:** Patient characteristics, according to asthma severity and body mass index

	Severe Asthma		Non-severe Asthma		*P*-value		
	BMI < 30 kg/m^2^ (*n* = 459)	BMI ≥ 30 kg/m^2^ (*n* = 423)	BMI < 30 kg/m^2^ (*n* = 97)	BMI ≥ 30 kg/m^2^ (*n* = 40)	BMI < 30 versus BMI ≥ 30	Severe versus Non-severe Asthma	Asthma Severity x Obesity Interaction
BMI (kg/m^2^) (*n* = 1,019)	25.2 (3.1)	37.0 (7.3)	25.4 (3.0)	35.4 (4.4)	**< 0.001**	0.574	0.058
Age (years) (*n* = 1,000)	54.6 (15.8)	54.8 (13.8)	52.5 (17.1)	58.0 (14.6)	0.116	0.302	0.073
Age at asthma onset (years) (*n* = 881)	20 (7, 35)	17 (5, 38)	16 (5, 37)	19 (6, 45)	0.571	0.879	0.309
Respiratory Medications							
ICS dose (BDP µg/day) (*n* = 1,018)	1600 (1000, 2000)	1800 (1000, 2000)	800 (400, 1000)	800 (450, 1000)	**0.008**	**< 0.001**	0.990
OCS dose (mg/day) (*n* = 981)	0 (0, 1)	0 (0, 4)	0 (0, 0)	0 (0, 0)	0.184	** 0.001**	0.746
Pack years of current and ex-smokers (*n* = 372)	10 (3, 29)	12 (4, 26)	3 (1, 13)	18 (7, 25)	0.154	**0.001**	0.072
Lung function							
Pre-bronchodilator FEV_1_%pred (*n* = 765)	65.6 (22.5)	64.2 (20.0)	81.7 (20.6)	77.0 (18.6)	0.315	**< 0.001**	0.519
Post-bronchodilator FEV_1_%pred (*n* = 702)	72.0 (23.4)	70.8 (20.2)	84.4 (21.9)	79.9 (16.6)	0.331	**< 0.001**	0.600
Pre-bronchodilator FVC %pred (*n* = 756)	85.1 (19.9)	78.8 (18.2)	91.3 (17.4)	93.0 (34.8)	**0.001**	**0.002**	0.212
Pre-bronchodilator %FEV_1_/FVC (*n* = 789)	60.0 (14.2)	64.0 (13.1)	70.7 (12.0)	66.6 (14.8)	**0.003**	**< 0.001**	**0.013**
Asthma control (ACQ) score (*n* = 902)	2.17 (1.27)	2.54 (1.32)	0.91 (0.89)	1.06 (1.20)	**< 0.001**	**< 0.001**	0.237
AQLQ score (*n* = 888)	4.76 (1.31)	4.33 (1.38)	5.88 (0.92)	5.74 (1.13)	**< 0.001**	**< 0.001**	0.137
Activity limitation domain	4.91 (1.33)	4.37 (1.39)	5.97 (1.01)	5.77 (1.13)	**< 0.001**	**< 0.001**	0.097
Symptoms domain	4.62 (1.42)	4.24 (1.47)	5.83 (0.96)	5.68 (1.22)	**0.001**	**< 0.001**	0.264
Emotional function domain	4.62 (1.63)	4.30 (1.76)	5.89 (1.16)	5.76 (1.43)	**0.018**	**< 0.001**	0.403
nvironmental stimuli domain	4.90 (1.52)	4.48 (1.64)	5.71 (1.08)	5.75 (1.31)	**0.005**	**< 0.001**	0.079
No. of exacerbations in previous year (*n* = 506)	0 (0, 3)	1 (0, 3)	0 (0, 0)	0 (0, 1)	0.122	**< 0.001**	0.848
Airway Inflammation							
Sputum %eosinophils (*n* = 118)	3.8 (1.1, 15.8)	1.5 (0.5, 6.0)	1.3 (0.3, 6.1)	1.0 (0.2, 1.5)	0.205	0.055	0.386
Sputum %neutrophils (*n* = 117)	22.7 (9.3, 50.3)	36.8 (19.3, 68.8)	17.9 (7.4, 35.4)	33.5 (13.5, 49.1)	**0.023**	0.512	0.879
FeNO (ppb) (*n* = 433)	28 (12, 50)	23 (12, 57)	23 (14, 37)	17 (15, 39)	0.113	0.396	0.364

*Definition of abbreviations*: *ACQ*  Asthma Control Questionnaire, *AQLQ*  Asthma-related Quality of Life Questionnaire, *BDP*  Beclomethasone dipropionate, *BMI* = body mass index, *FeNO*  Fractional exhaled nitric oxide, *FEV1*  Forced expiratory volume in 1 s, *FVC*  Forced vital capacity, *ICS*  Inhaled corticosteroids, *OCS*  Oral corticosteroids

*P*-values adjusted for asthma severity, obesity, inhaled corticosteroid dose (beclomethasone dipropionate equivalents/day), oral corticosteroid dose (mg/day), biologic use (yes/no) and smoking status (never/ex/current), except in models where that particular variable is the dependent variable

Patients with severe asthma had a lower pre-bronchodilator %predicted FEV_1_ than patients with non-severe asthma, however there was no impact of obesity on %predicted FEV_1_ (Fig. 1c; Table 2, Table S4). Patients with *versus* without obesity had a lower %predicted FVC [MD (95% CI) = -5.2 (-8.3 to -2.1)%, *p* = 0.001]. Similarly, patients who had severe asthma *versus* non-severe asthma also had a lower %predicted FVC [MD (95% CI) = -8.4 (-13.8 to -2.1)%, *p* = 0.002] (Fig. 1d; Table 2, Table S4). There was a significant interaction between obesity and asthma severity, whereby patients who had both obesity and severe asthma had a higher FEV_1_/FVC (*p* = 0.031, Table 2, Table S4). This result was driven by the lower FVC, but not FEV_1_, observed in patients with both severe asthma and obesity.

**Fig. 2:**
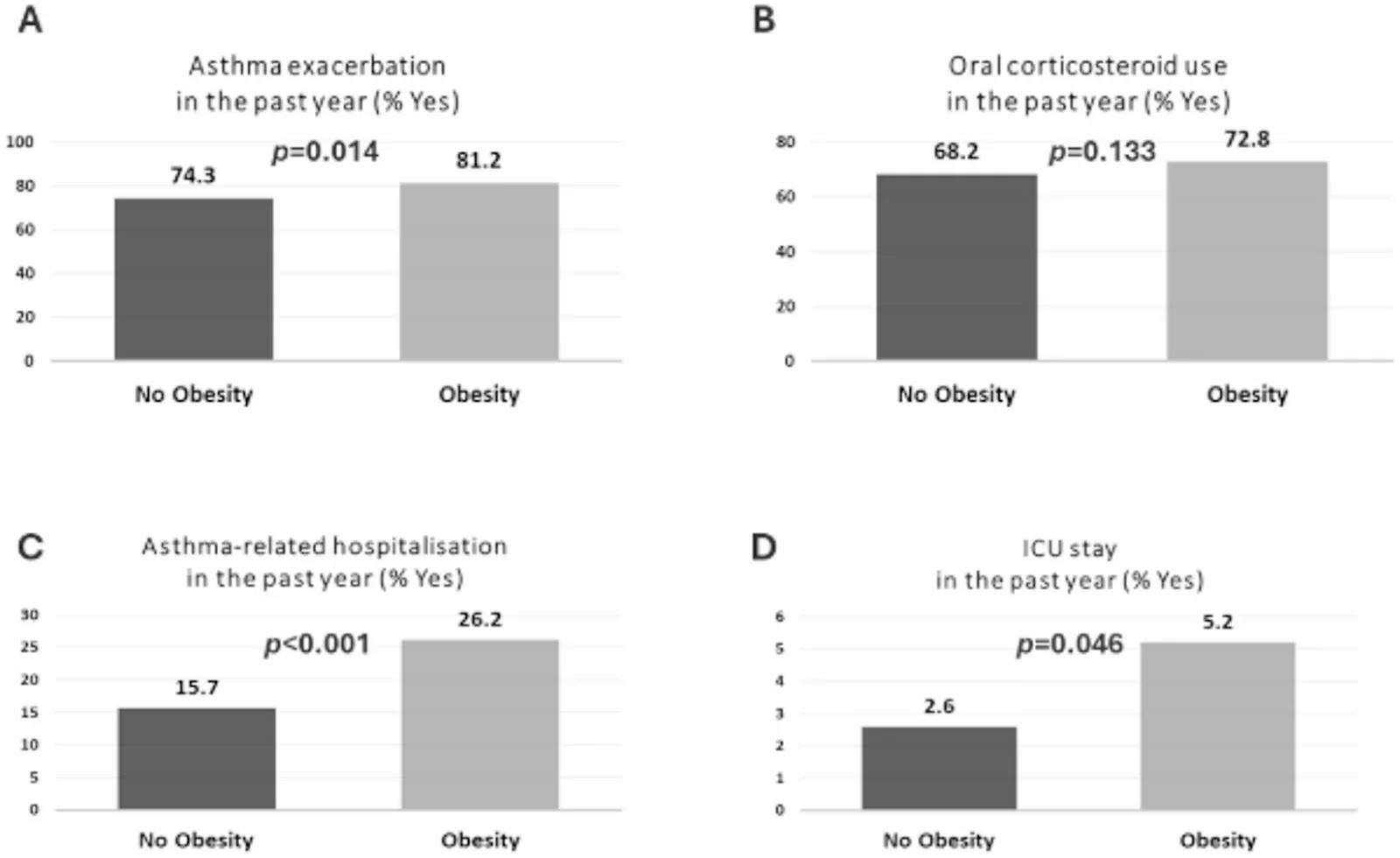
Proportion of patients with severe asthma without *versus* with obesity, who in the past year had: (**a**) an asthma exacerbation, (**b**) used oral corticosteroids, (**c**) an asthma-related hospitalisation, and (**d**) an ICU stay

A higher proportion of patients with severe asthma and obesity had experienced an asthma exacerbation in the previous 12 months compared with patients with severe asthma but without obesity (81.2% vs. 74.3%, *p* = 0.014; Fig. 2; Table 3). This included 1.67 times the odds of hospitalisation for asthma, 2.00 times the odds of ICU admission for asthma, and 1.35 times the odds of an unscheduled doctor’s visit for asthma (Fig. 2; Table 3). In the non-severe asthma group, the presence of obesity quadrupled the odds of hospitalisation for asthma (12.5% vs. 3.1%, *p* < 0.001) (Table 3).

**Table 3:** Categorical patient characteristics, according to asthma severity and body mass index

	Severe Asthma			Non-severe Asthma			*P*-value*
	BMI < 30 kg/m^2^	BMI ≥ 30 kg/m^2^	*P*-value	BMI < 30 kg/m^2^	BMI ≥ 30 kg/m^2^	*P*-value	
Sex (n, %female) (*n* = 1,017)	270/457 (59.1%)	281/423 (66.4%)	**0.024**	51/97 (52.6%)	28/40 (70.0%)	0.061	**0.019**
Respiratory Medications							
Daily oral corticosteroid use	116/451 (25.7%)	113/409 (27.6%)	0.527	3/95 (3.2%)	2/38 (5.3%)	0.564	**< 0.001**
Anti-IgE (Omalizumab)	56/448 (12.5%)	54/401 (13.5%)	0.676	0/94 (0.0%)	0/37 (0.0%)	-	**< 0.001**
Anti-IL-5 (Mepolizumab/Benralizumab)	102/453 (22.5%)	98/417 (23.5%)	0.730	0/94 (0.0%)	0/37 (0.0%)	**-**	**< 0.001**
Anti-IL-4 (Dupilumab)	11/448 (2.5%)	8/406 (2.0%)	0.631	0/94 (0.0%)	0/37 (0.0%)	-	0.357
Exacerbation in previous year (n, %)	341/459 (74.3%)	341/420 (81.2%)	**0.014**	38/97 (39.2%)	22/39 (56/4%)	0.067	**< 0.001**
Hospitalised (n, %)	72/459 (15.7%)	111/423 (26.2%)	**< 0.001**	3/97 (3.1%)	5/40 (12.5%)	**< 0.001**	**< 0.001**
Intensive Care Unit stay (n, %)	12/459 (2.6%)	22/423 (5.2%)	**0.046**	1/97 (1.0%)	1/40 (2.5%)	0.515	0.090
Visited emergency room (n, %)	71/459 (15.5%)	86/423 (20.3%)	0.059	4/97 (4.1%)	2/40 (5.0%)	0.820	**< 0.001**
Unscheduled doctor visit (n, %)	142/459 (30.9%)	176/423 (41.6%)	**0.001**	22/97 (22.7%)	11/40 (27.5%)	0.549	**< 0.001**
Oral corticosteroids prescribed (n, %)	313/459 (68.2%)	308/423 (72.8%)	0.133	31/97 (32.0%)	19/40 (47.5%)	0.086	**< 0.001**
Airway Inflammation							
Eosinophilic airway inflammation (n, %)	111/203 (54.7%)	84/185 (45.4%)	0.068	23/54 (42.6%)	7/22 (31.8%)	0.383	0.071
Neutrophilic airway inflammation (n, %)	6/44 (13.6%)	11/44 (25.0%)	0.177	0/20 (0.0%)	1/8 (12.5%)	0.107	0.076

*p-value for overall difference between groups

### Exploring airway inflammation and sex as potential drivers of poorer asthma outcomes in obesity-related asthma

Patients with severe asthma and obesity were more likely to be female compared with patients with severe asthma without obesity (66.4% vs. 59.1%, *p* = 0.024; Table 3). There was a similar pattern in non-severe asthma, however this did not reach statistical significance (*p* = 0.061).

**Fig. 3:**
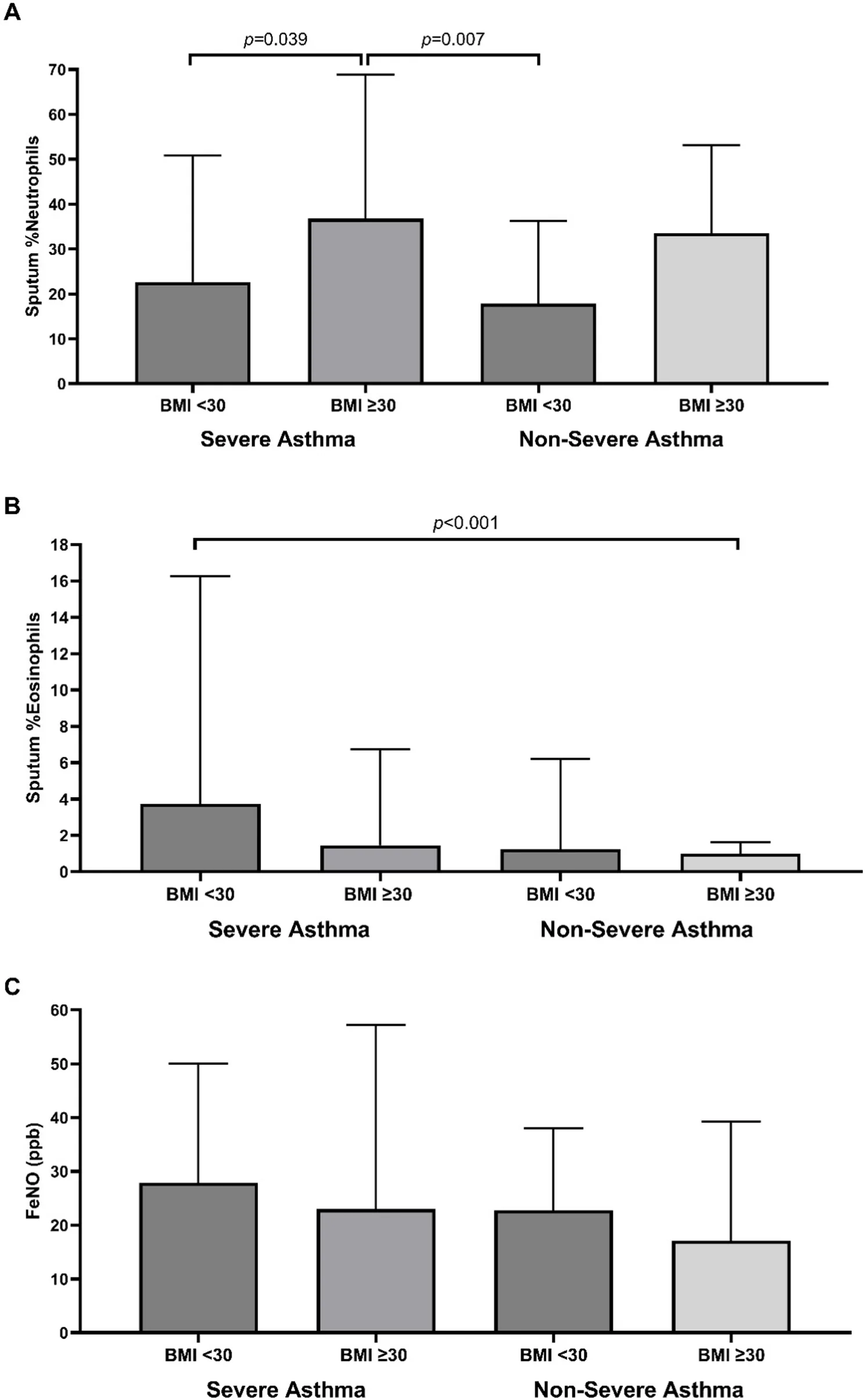
Median (IQR) (**a**) sputum %neutrophils, (**b**) sputum %eosinophils, and (**c**) fractional exhaled nitric oxide (FeNO), according to asthma severity and body mass index (BMI)

**Fig. 4:**
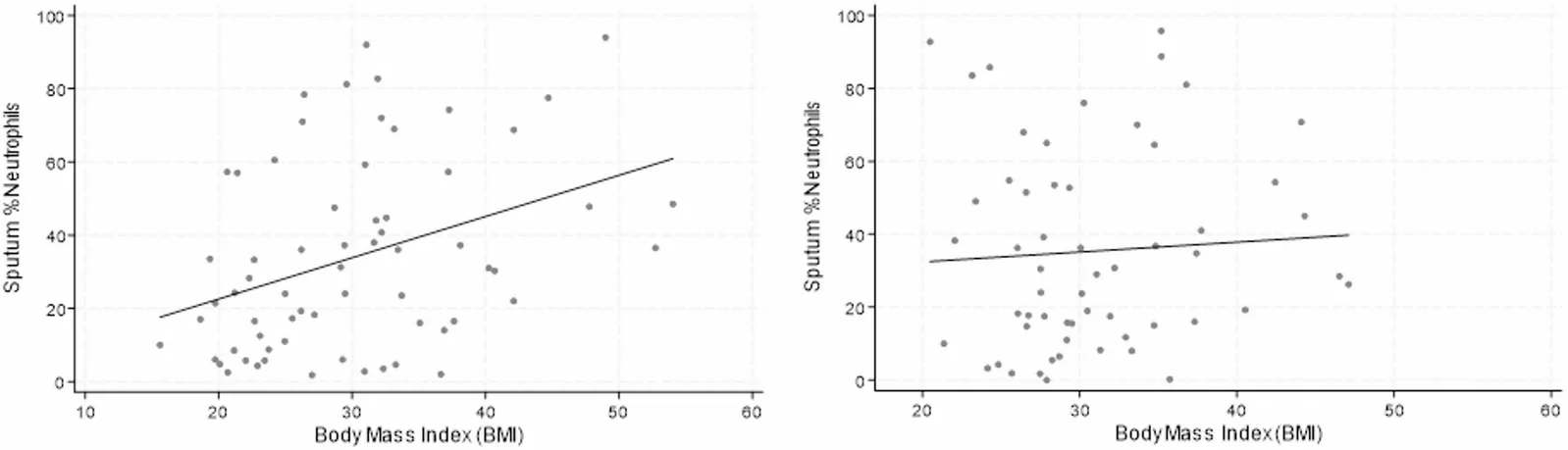
Correlation between body mass index (BMI) and sputum %neutrophils in (**a**) females (*r*_*s*_=0.370, *p* = 0.003), and (**b**) males (*r*_*s*_=0.092, *p* = 0.495)

Baseline characteristics were compared in patients who had sputum samples collected compared with those who did not (Table S5). Patients living with obesity had higher sputum %neutrophils than patients without obesity [MD (95% CI) = 12.1 (1.7 to 22.4)%, *p* = 0.023] (Table 2, Table S4). When examined by asthma severity, this effect was more pronounced in patients with severe asthma (Fig. 3a). Sputum %neutrophils were positively correlated with BMI (*r*_*s*_=0.256, *p* = 0.005). Interestingly, this correlation was apparent in females (*r*_*s*_=0.370, *p* = 0.003) but not males (*r*_*s*_=0.092, *p* = 0.495) (Fig. 4). Women with obesity had higher sputum %neutrophils than women without obesity [39.4 (22.0, 68.8)% vs. 19.3 (8.5, 36.0)%, *p* = 0.013]. Conversely, there was no significant difference in sputum %neutrophils in men with obesity compared with men without obesity [30.8 (17.5, 64.5)% vs. 21.1 (10.0, 52.8)%, *p* = 0.254]. Sputum %neutrophils correlated with worse asthma control (*r*_*s*_=0.254, *p* = 0.005) and lower FEV_1_%predicted (*r*_*s*_=-0.356, *p* < 0.001), FVC %predicted (*r*_*s*_=-0.321, *p* < 0.001) and FEV_1_/FVC (*r*_*s*_=-0.193, *p* = 0.044).

There was no difference in sputum %eosinophils in patients with *versus* without obesity (*p* = 0.205) (Table 2, Table S4, Fig. 3b), and there was no correlation between sputum %eosinophils and BMI (*r*_*s*_=-0.123, *p* = 0.177), asthma control, or lung function parameters. FeNO did not differ according to asthma severity (*p* = 0.396) or the presence of obesity (*p* = 0.113) (Fig. 3c). There was no correlation between FeNO and BMI (*r*_*s*_=0.005, *p* = 0.915).

### Effect of obesity on the presence of other comorbidities

Obesity was associated with a higher prevalence of recorded OSA, diabetes and GORD, in both severe and non-severe asthma (Table S6). Patients with severe asthma and obesity also had a higher prevalence of anxiety and depression, and a lower prevalence of allergic bronchopulmonary aspergillosis, compared with patients with severe asthma who did not have obesity (Figure S1; Table S6).

### Longitudinal changes in clinical outcomes in patients with severe asthma after 12 months of biologic medication

Patients with severe asthma and obesity who were prescribed biologic medication lost weight over the 12-month follow-up period [-2.3 (5.3)kg] (Table 4). Interestingly, weight loss was also observed in patients with severe asthma and obesity who were not prescribed biologic medication [-1.7 (6.7)kg] (Table 4). There was a significant reduction in daily OCS dose in patients with obesity prescribed biologic medication [-4.3 (10.6)mg/day, *p* = 0.018], while there was no significant change in daily OCS dose in patients prescribed a biologic who did not have obesity [-0.6 (6.1), *p* = 0.551] (Figure S2). There were statistically significant improvements in both ACQ and AQLQ in patients with and without obesity prescribed biologic medication (Figure S2). There was also a small improvement in these parameters in patients not prescribed biologic medication (Table 4). While there was a significant improvement in lung function in patients prescribed biologic medication without obesity (pre- and post-bronchodilator FEV_1_, FVC and FEV_1_/FVC), there was no change in lung function in patients with obesity prescribed biologic medication (Figure S2; Table 4).

**Table 4:** Changes in patient characteristics at 12 months in adults with severe asthma, according to biologic medication use and BMI

	Using Biologic Medication at 12 Months			Not Using Biologic Medication at 12 Months			*P*-value §
	BMI < 30 kg/m^2^ (*n* = 48)	BMI ≥ 30 kg/m^2^ (*n* = 37)	*P*-value	BMI < 30 kg/m^2^ (*n* = 125)	BMI ≥ 30 kg/m^2^ (*n* = 111)	*P*-value	
Weight (kg) (*n* = 321)	1.1 (5.9)	-2.3 (5.3) *†	**0.008**	-0.1 (3.9) ‡	-1.7 (6.7) *†	**0.024**	**0.004**
Pre-bronchodilator FEV_1_ (L) (*n* = 169)	0.24 (0.09, 0.67) *	0.05 (-0.11, 0.19) †	**0.003**	-0.02 (-0.17, 0.09) †	0.10 (-0.07, 0.18) *†	**0.022**	**< 0.001**
Pre-bronchodilator FEV_1_%pred (*n* = 156)	8.8 (3.2, 18.0) *	1.6 (-2.0, 7.7) †	**0.013**	1.2 (-3.6, 5.5) †	3.4 (-2.8, 7.9) †	0.348	**0.002**
Post-bronchodilator FEV_1_ (L) (*n* = 138)	0.17 (0.01, 0.37) *	0.04 (-0.04, 0.22)	0.252	-0.04 (-0.09, 0.07) †	-0.01 (-0.16, 0.13) †	0.695	**0.006**
Post-bronchodilator FEV_1_%pred (*n* = 133)	5.3 (0.1, 12.0) *	1.4 (-1.0, 6.2)	0.317	0.3 (-3.1, 4.0)	-0.5 (-6.3, 5.4)	0.576	0.052
Pre-bronchodilator FVC (L) (*n* = 168)	0.34 (0.49) *	-0.02 (0.46) †	**0.014**	-0.04 (0.46) †	0.08 (0.37) †	0.120	**0.002**
Pre-bronchodilator FVC %pred (*n* = 155)	7.5 (16.3) *	2.2 (11.4)	0.242	0.9 (12.9)	2.3 (17.0)	0.640	0.297
Pre-bronchodilator %FEV_1_/FVC (*n* = 168)	5.1 (13.0) *	0.2 (9.0)	0.152	-0.2 (6.4)	0.9 (8.1)	0.417	0.058
Asthma control (ACQ) score (*n* = 277)	-1.75 (1.30) *	-1.82 (1.52) *	0.838	-0.35 (1.16) *†‡	-0.35 (1.02) *†‡	0.970	**< 0.001**
AQLQ score (*n* = 270)	1.53 (1.43) *	0.92 (1.28) *	0.072	0.32 (1.12) *†‡	0.38 (1.03) *†‡	0.681	**< 0.001**
Activity limitation domain	1.26 (1.56) *	0.87 (1.33) *	0.278	0.28 (1.13) *†‡	0.29 (1.12) *†‡	0.937	**< 0.001**
Symptoms domain	1.66 (1.58) *	0.94 (1.33) *	0.047	0.37 (1.26) *†	0.46 (1.14) *†	0.577	**< 0.001**
Emotional function domain	1.62 (1.72) *	0.87 (1.53) *	0.063	0.37 (1.43) *†	0.49 (1.40) *†	0.548	**< 0.001**
Environmental stimuli domain	1.29 (1.98) *	1.07 (1.52) *	0.613	0.25 (1.30) †‡	0.27 (1.23) *†‡	0.910	**< 0.001**
Oral corticosteroid dose (mg/day)	-0.6 (6.1)	-4.3 (10.6) *	0.377	1.2 (10.6)	-1.6 (6.2) *	0.135	0.224
No. of exacerbations in previous year (*n* = 111)	-2 (-4, 0) *	-3 (-4, -1) *	0.181	0 (0, 0) †‡	0 (0, 0) †‡	0.677	**< 0.001**
FeNO (ppb) (*n* = 65)	-1 (-25, 11)	-10 (-39, 12)	0.609	1 (-3, 9)	-3 (-18, 4)	0.268	0.664

Data presented as mean (SD) or median (IQR)

*ACQ*  Asthma Control Questionnaire, *AQLQ*  Asthma-related Quality of Life Questionnaire, *BD* Bronchodilator, *FeNO* Fractional exhaled nitric oxide, *FEV*_1_ Forced Expiratory Volume in 1-second, *FVC* Forced Vital Capacity

* *p* < 0.05 at baseline *versus* at 12 months

† *p* < 0.017 *versus* patients using biologic medication at 12 months without obesity

‡ *p* < 0.017 *versus* patients using biologic medication at 12 months with obesity

§ *p*-value for overall difference between groups

## Discussion

In this real-world study, we demonstrate that severe asthma is associated with an increased prevalence of a range of comorbidities including obesity, OSA, nasal polyps, bronchiectasis and GORD. We also show that the presence of obesity further increases the prevalence of a range of comorbidities in adults with severe asthma, including OSA, diabetes, anxiety, depression, and GORD. Our data demonstrates that obesity is associated with worse respiratory outcomes in patients with severe asthma, including asthma control, asthma-related quality of life and lung function, as well as an increased odds of experiencing an asthma exacerbation, including hospitalisation and intensive care admission for asthma. Importantly, we show that a higher BMI is associated with sputum neutrophilia in women, but not in men, and that airway neutrophils correlate with worse asthma control and lung function.

Obesity is a known risk factor for developing asthma that is more severe and difficult to treat, and is associated with a reduced quality of life [23]. In line with our observations, other studies of registry data also demonstrate a high prevalence of obesity in the “difficult” asthma group, with increased medication requirements and a higher prevalence of comorbidities [24, 25]. The increased risk of comorbidities may be due to a number of factors including increases in mechanical forces associated with excess adiposity that may influence OSA, or altered metabolic processes widely known to be associated with diabetes, GORD, cardiovascular disease and stroke [26]. Obesity is also known to impact immune cell function, which increases susceptibility to infectious exacerbations and may contribute to more frequent unplanned doctor visits and hospital admissions [27]. 

Hypothesis-free cluster analyses have identified an obesity-related asthma phenotype that is characterised by obesity, female-predominance, late onset asthma, neutrophilic or mixed granulocytic airway inflammation, and high-dose ICS therapy [13, 28–32]. In support of this, a meta-analysis demonstrated that sputum neutrophils are 5% higher in adults with asthma and obesity compared with those with asthma but without obesity [33]. Our findings extend on this, as we show in a real-world setting that patients with comorbid asthma and obesity are more likely to be female, have a higher ICS dose, and that the obesity-induced increase in sputum neutrophils is only evident in females. The mechanisms driving this increased neutrophilic airway inflammation in women with obesity is unknown, but it is possible that sex hormones and adiposity may have an interconnected role, as obesity is linked with sex hormone abnormalities [34, 35]. Australian data demonstrates that women are twice as likely to die from asthma than men [36]. In pre-menopausal women, up to half of emergency room presentations for asthma occur during the late luteal phase of the menstrual cycle, comprising the days immediately prior to menstruation [37]. In post-menopausal women, menopausal hormone therapy, consisting of estrogen or estrogen plus progestogen, has been linked with an increased asthma incidence and wheeze [38]. Excess adiposity also leads to inflammasome activation, which upregulates IL-1β release leading to neutrophilic airway inflammation [39, 40]. Taken together, this suggests a mechanism through which women with obesity are more susceptible to neutrophilic airway inflammation and asthma-related mortality.

Lung function impairment is known to occur in both asthma and obesity [41], and determining the relative contribution of each is challenging. Small airway dysfunction is associated with severe asthma and is related to airway remodelling and air trapping, which also occurs in obesity [41]. Additionally, lung and thoracic compliance is affected by obesity and influences lung function. Accumulation of adipose tissue in the thoracic and abdominal cavities impairs diaphragmatic and chest wall movement, reduces thoracic compliance and increases pleural pressure, with resultant restrictive ventilatory defect with reduction in functional residual capacity [42–44]. The effect on total lung capacity and residual volume is modest even in severe obesity [43], but expiratory reserve volume is reduced. In our cohort, FEV_1_ was influenced by asthma severity but not obesity, whilst FVC was reduced by both asthma severity and obesity. An inverse association between obesity and FVC was also identified in a Norwegian study of people with non-severe asthma [45]. FVC can be decreased by air trapping, airway remodelling, and reduced thoracic compliance [46, 47], all of which could be reasonably encountered in people with obesity and asthma. This observation in our cohort appears to be due to a more marked reduction in FVC in those with severe asthma and obesity, as those with obesity and non-severe asthma demonstrated a generally preserved FVC. This may be partly explained by the fact that patients with severe asthma had a higher prevalence of morbid obesity, however it is likely there are also other factors involved.

Our longitudinal data shows that patients with severe asthma and obesity prescribed biologic medication experienced significant weight loss at 12 months, whereas patients prescribed a biologic who did not have obesity did not lose weight. Another study, which also observed weight loss in patients with asthma and obesity after being prescribed biologic medication, postulated this could be explained by a reduction in daily OCS use [48]. Our data shows a significant 4 mg/day reduction in OCS dose in patients with obesity who commenced biologic medication, while there was no change in daily OCS in patients prescribed a biologic who did not have obesity. We hypothesise that improved diet quality and increased energy expenditure in the setting of reduced asthma symptoms may also contribute to these observations. Although we were unable to examine this directly in our study, our hypothesis is supported by our observation that patients with severe asthma and obesity not prescribed biologic medication also experienced a reduction in asthma symptoms that corresponded with a small reduction in weight.

Our study confirms that people with severe asthma and obesity benefit from biologic therapy. While there was no change in lung function in patients with obesity, there was a significant reduction in asthma exacerbations and OCS use, and improvements in asthma symptoms and asthma-related quality of life. In line with our observations, a meta-analysis of the MENSA and MUSCA trials also observed biologic use was associated with a reduction in exacerbations in patients with obesity, while there was no change in lung function [49]. Longitudinal data from the UK Severe Asthma Registry supports our findings, with patients with obesity across the range of BMI categories demonstrating improvements in asthma control, but not in lung function [50]. In contrast, Oliveira [51] did observe improvements in FEV_1_ in patients with obesity after 12 months of omalizumab, however these patients also lost 5% body weight. This highlights the need to conduct research examining whether weight loss augments the beneficial effects of biologic therapy in patients with obesity.

A strength of this study is that it is the first to detail differences in a wide range of clinical and inflammatory asthma outcomes in a real-world cohort of patients with and without obesity, recruited across 28 sites in Australia and New Zealand. Another strength is that we were able to examine airway inflammation as a potential driver of the poorer outcomes observed in obesity-related asthma, including sex-based differences. Additionally, regression modelling was adjusted for potential confounders including ICS dose, OCS use, biologic use, and smoking history, all of which may plausibly influence body weight, inflammatory profiles, exacerbation frequency, and lung function. A limitation is that we did not have data on body composition (adipose tissue quantity and distribution), dietary intake, metabolic health, microbiomes and metabolomes, sex hormones, and numerous molecular and cellular factors that may have an impact. Additionally, there was only sputum data available for a subset (117) of patients, which may affect the generalisability of the findings. Nonetheless, this data provides important insight into the comorbidities experienced by patients with severe and non-severe asthma, including obesity, and demonstrates for the first time in a real-world setting that the previously discovered elevation in neutrophilic airway inflammation observed in adults with obesity-related asthma is only apparent in females.

## Conclusions

In conclusion, severe asthma is associated with an increased risk of a number of comorbidities, including obesity. Our real-world data clearly demonstrates that obesity is associated with more severe disease, neutrophilic airway inflammation, increased exacerbation risk, and other comorbidities. We observed that neutrophilic airway inflammation is higher in women with obesity, but not men with obesity, highlighting a potential mechanism to explain the increased asthma severity observed in females with asthma. We also show that biologic treatment is effective in reducing asthma symptoms and exacerbations in patients with severe asthma and obesity, despite no effect on lung function; thus supporting its efficacy in people with severe asthma and obesity. Given the poorer outcomes observed in this population, targeted treatment of obesity as part of the management of asthma may provide additional benefit beyond standard asthma care. More research is warranted to better understand the sex-based differences in obesity-related asthma as well as the ideal treatment approach for this population.

## Supplementary Information

Supplementary Material 1.

## Data Availability

The data that support the findings of this study are available from the Australasian Severe Asthma Registry (ASAR) but restrictions apply to the availability of these data, which were used under license for the current study, and so are not publicly available. Data are however available from the authors upon reasonable request and with permission of the ASAR.

## Abbreviations

ACQ: Asthma Control Questionnaire
ACT: Asthma Control Test
AQLQ: Asthma Quality of Life Questionnaire
ASAR: Australasian Severe Asthma Registry
BMI: Body Mass Index
COPD: Chronic Obstructive Pulmonary Disease
FeNO: Fractional Exhaled Nitric Oxide
FEV_1_: Forced Expiratory Volume in 1-second
FVC: Forced Vital Capacity
GORD: Gastro-Oesophageal Reflux Disease
ICS: Inhaled Corticosteroids
OCS: Oral Corticosteroids
ICU: Intensive care unit
OSA: Obstructive Sleep Apnoea
VAO: Variable Airflow Obstruction
VCD: Vocal Cord Dysfunction
