# Supplementary material for: Relationship between asthma, obesity and other comorbidities: the Australasian Severe Asthma Registry
Source: Respir Res. 2026 Aug 21;27:377. doi: 10.1186/s12931-026-03872-3 (PMC13628815; doi:10.1186/s12931-026-03872-3)
Supplement: Supplementary file 1 [file 12931_2026_3872_MOESM1_ESM.docx]

**Relationship between asthma, obesity and other comorbidities: the Australasian Severe Asthma Registry**

**Authors**

Hayley A Scott, PhD,^a,b^ Joy Lee, MBChB, PhD,^c,d,e^ Eve Denton, MD, PhD,^d,e^ Adrian Barnett, MD,^f^ Paul Griffiths, MD,^g^ Peter G Middleton, MD, PhD,^h,i^ Philip M Hansbro, PhD^j^

**Institutional Affiliations**

^a^ School of Biomedical Sciences and Pharmacy, The University of Newcastle, University Drive, Callaghan, NSW, Australia.

^b^ Immune Health Research Program, Hunter Medical Research Institute, Lot 1 Kookaburra Circuit, New Lambton Heights, NSW, Australia.

^c^ Department of Respiratory and Sleep Medicine, Austin Health, Melbourne VIC, Australia.

^d^ School of Translational Medicine, Monash University, Melbourne, VIC, Australia.

^e^ Asthma, Allergy and Clinical Immunology, Respiratory Medicine, Alfred Hospital, Melbourne, VIC, Australia.

^f^ Department of Respiratory and Sleep Medicine, Mater Hospital, South Brisbane, QLD, Australia.

^g^ Department of Respiratory Medicine, North Shore Hospital, Auckland, New Zealand.

^h^ Department of Respiratory and Sleep Medicine, Westmead Hospital, NSW, Australia.

^i^ Ludwig Engel Centre for Respiratory Research, The Westmead Institute for Medical Research, NSW, Australia.

^j^ Centre for Inflammation, Centenary Institute and University of Technology Sydney, Faculty of Science, School of Life Sciences, Sydney, NSW, Australia.

**Corresponding Author**

Dr Hayley Scott, hayley.scott@newcastle.edu.au,

Phone: +61 2 4042 0113, Fax: +61 2 4042 0046

Immune Health Research Program, Level 2 West, Hunter Medical Research Institute.

Lot 1 Kookaburra Circuit, New Lambton Heights NSW 2305, Australia.

**Appendix E1 –** *Supplementary Methods*

**Patients**

Patients were ≥18 years old and had confirmed asthma demonstrated by variable airflow obstruction (VAO), either at their first visit or documented within the past 10 years. VAO was demonstrated by either bronchodilator response (>200ml and/or 12% post 400mcg salbutamol), airway hyperresponsiveness (AHR) to bronchial provocation challenge agent, peak flow variability (>12% over 1 week) or variability in forced expiratory volume in 1-second (FEV_1_, >12% within last two months). Patients with severe asthma were on the highest level of recommended treatment [maximal inhaled corticosteroid (ICS) therapy with second controller] and had poor asthma control, as manifested by one or more of the following^17^:

- Poor symptom control: asthma control questionnaire (ACQ6) >1.5, asthma control test ACT <20);
- Frequent, severe exacerbations (>/2 bursts of systemic corticosteroids >3 days in the last 12 months;
- Serious exacerbation (hospitalisation, intensive care unit ICU stay or mechanical ventilation in the last 12months); and/or
- Persistent airflow limitation (FEV_1_<80% predicted with reduced FEV_1_/forced vital capacity, FVC).

The comparison group was adults with non-severe asthma who were also prescribed maintenance inhaled controller therapy and had partially-to-well controlled asthma, defined as an ACQ6 <1.5^18^ or ACT >20,^19^ and stable disease with no history of infection, exacerbation or change in preventer therapy in the previous 4 weeks. Patients with cognitive impairment, highly dependent on medical care, an inability to attend study visits, other primary lung disease, significant life-limiting comorbidity, or any history of malignancy were excluded. Patients who had a current asthma exacerbation were not enrolled during the exacerbation, but were eligible to be enrolled when stable.

**Supplementary Tables**

**Table S1.** Clinical asthma and inflammatory characteristics, according to asthma severity.

| **Characteristic** | **Severe asthma (n=882)** | **Non-severe asthma (n=137)** | **P-value** |
| --- | --- | --- | --- |
| Lung function |  |  |  |
| Pre-bronchodilator FEV_1_ %pred (n=765) | 64.9 (21.4) | 80.4 (20.1) | **<0.001** |
| Post-bronchodilator FEV_1_ %pred (n=702) | 71.5 (21.9) | 83.2 (20.6) | **<0.001** |
| Pre-bronchodilator FVC %pred (n=756) | 82.1 (19.3) | 91.8 (23.3) | **<0.001** |
| Pre-bronchodilator %FEV_1_/FVC (n=789) | 62.0 (13.8) | 69.6 (12.9) | **<0.001** |
| Asthma control (ACQ6) score (n=902) | 2.35 (1.31) | 0.95 (0.98) | **<0.001** |
| AQLQ score (n=888) | 4.55 (1.26) | 5.84 (0.99) | **<0.001** |
| Activity limitation domain | 4.65 (1.38) | 5.92 (1.04) | **<0.001** |
| Symptoms domain | 4.43 (1.46) | 5.79 (1.04) | **<0.001** |
| Emotional function domain | 4.46 (1.70) | 5.85 (1.24) | **<0.001** |
| Environmental stimuli domain | 4.70 (1.59) | 5.72 (1.14) | **<0.001** |
| Exacerbation in previous year (n, %) | 682/879 (77.6%) | 60/136 (44.1%) | **<0.001** |
| No. of exacerbations in previous year (n=506) | 1 (0, 3) | 0 (0, 0) | **<0.001** |
| Hospitalised (n, %) | 183/882 (20.8%) | 8/137 (5.8%) | **<0.001** |
| Intensive care unit stay (n, %) | 34/882 (3.9%) | 2/137 (1.5%) | 0.158 |
| Visited emergency room (n, %) | 157/882 (17.8%) | 6/137 (4.4%) | **<0.001** |
| Unscheduled doctor visit (n, %) | 318/882 (36.1%) | 33/137 (24.1%) | **0.006** |
| Oral corticosteroids prescribed (n, %) | 621/882 (70.4%) | 50/137 (36.5%) | **<0.001** |
| Airway inflammation |  |  |  |
| Sputum eosinophils (%) (n=118) | 2.5 (0.5, 12.3) | 1.1 (0.3, 4.5) | **0.039** |
| Eosinophilic asthma (n, %) | 195/388 (50.3%) | 30/76 (39.5%) | 0.085 |
| Sputum neutrophils (%) (n=116) | 31.0 (15.5, 57.3) | 19.1 (8.4, 40.0) | 0.129 |
| Neutrophilic asthma (n, %) | 17/88 (19.3%) | 1/28 (3.6%) | **0.045** |
| FeNO (ppb) (n=433) | 25 (12, 54) | 22 (14, 39) | 0.259 |

Definition of abbreviations: ACQ = Asthma Control Questionnaire; AQLQ = Asthma-related Quality of Life Questionnaire; FeNO = fractional exhaled nitric oxide; FEV_1_ = forced expiratory volume in 1 second; FVC = forced vital capacity.

**Table S2.** Presence of pulmonary and extrapulmonary comorbidities, according to asthma severity.

| **Comorbidity (n, %)** | **Severe asthma**  **(n=882)** | **Non-severe asthma (n=137)** | **P-value** |
| --- | --- | --- | --- |
| **Pulmonary and upper airways** |  |  |  |
| Allergic rhinitis | 437 (49.6%) | 68 (49.6%) | 0.985 |
| Chronic rhinosinusitis | 78 (8.8%) | 8 (5.8%) | 0.239 |
| Nasal polyps | 169 (19.2%) | 14 (10.2%) | **0.011** |
| Vocal cord dysfunction | 58 (6.6%) | 4 (2.9%) | 0.096 |
| Obstructive sleep apnea | 139 (15.8%) | 10 (7.3%) | **0.009** |
| Bronchiectasis | 68 (7.7%) | 4 (2.9%) | **0.042** |
| Chronic obstructive pulmonary disease | 83 (9.4%) | 5 (3.7%) | **0.026** |
| Hyperventilation dysfunctional breathing | 20 (2.3%) | 1 (0.7%) | 0.239 |
| Allergic bronchopulmonary aspergillosis | 42 (4.8%) | 3 (2.2%) | 0.173 |
| **Extra-pulmonary** |  |  |  |
| Metabolic disease |  |  |  |
| Obesity | 423 (48.0%) | 40 (29.2%) | **<0.001** |
| Diabetes | 108 (12.2%) | 11 (8.0%) | 0.153 |
| Cardiovascular disease |  |  |  |
| Myocardial infarction | 20 (2.3%) | 3 (2.2%) | 0.955 |
| Stroke | 6 (0.7%) | 2 (1.5%) | 0.336 |
| Other cardiovascular disease | 166 (18.8%) | 41 (29.9%) | **0.003** |
| Psychiatric disease |  |  |  |
| Anxiety | 154 (17.5%) | 19 (13.9%) | 0.298 |
| Depression | 177 (20.1%) | 22 (16.1%) | 0.271 |
| Gastro-oesophageal reflux disease (GORD) | 376 (42.6%) | 45 (32.9%) | **0.030** |
| Osteoporosis | 118 (13.4%) | 11 (8.0%) | 0.080 |
| Allergic disease |  |  |  |
| Eczema | 146 (16.6%) | 26 (19.0%) | 0.481 |
| Anaphylaxis | 61 (6.9%) | 8 (5.8%) | 0.641 |

# **Table S3.** Prevalence of comorbidities that are being actively managed.

| **Comorbidity** | **Severe asthma** | **Non-severe asthma** | **P-value** |
| --- | --- | --- | --- |
| Allergic rhinitis | 362/412 (87.9%) | 49/62 (79.0%) | 0.056 |
| Nasal polyps | 141/160 (88.1%) | 12/13 (92.3%) | 0.650 |
| Vocal cord dysfunction | 35/47 (74.5%) | 3/4 (75.0%) | 0.981 |
| Obstructive sleep apnoea | 108/128 (84.4%) | 8/9 (88.9%) | 0.716 |
| Bronchiectasis | 55/59 (93.2%) | 2/3 (66.7%) | 0.099 |
| COPD | 72/75 (96.0%) | 4/4 (100.0%) | 0.683 |
| GORD | 298/316 (94.3%) | 24/31 (77.4%) | 0.001 |

Definition of abbreviations**:** COPD = chronic obstructive pulmonary disease; GORD: gastro-oesophageal reflux disease.

**Table S4.** Mean differences in patient characteristics, according to asthma severity and obesity status.

|  | **BMI ≥30 *versus* BMI <30** | | **Severe *versus* Non-severe Asthma** | | **Obesity x Asthma Severity Interaction** |
| --- | --- | --- | --- | --- | --- |
|  | **Mean Difference (95% CI)** | **P-value** | **Mean Difference (95% CI)** | **P-value** |  |
| Age (years) (n=934) | 1.5 (-0.4 to 3.5) | 0.116 | 1.7 (-1.5 to 3.5) | 0.302 | 0.073 |
| Age at asthma onset (years) (n=849) | -0.8 (-3.4 to 1.9) | 0.571 | 0.3 (-3.4 to 4.6) | 0.879 | 0.309 |
| Respiratory Medications |  |  |  |  |  |
| ICS dose (BDP µg/day) (n=952) | 157 (41, 274) | **0.008** | 971 (861 to 1082) | **<0.001** | 0.990 |
| OCS dose (mg/day) (n=952) | 0.6 (-0.3 to 1.5) | 0.184 | 1.6 (0.7 to 2.4) | **0.001** | 0.746 |
| Pack years of current and ex-smokers (n=353) | 3.1 (-1.2 to 7.4) | 0.154 | 8.9 (3.9 to 13.9) | **0.001** | 0.072 |
| Lung function |  |  |  |  |  |
| Pre-bronchodilator FEV_1_ %pred (n=733) | -1.6 (-4.7 to 1.5) | 0.315 | -13.8 (-18.5 to -9.1) | **<0.001** | 0.519 |
| Post-bronchodilator FEV_1_ %pred (n=673) | -1.6 (-5.0 to 1.7) | 0.331 | -10.7 (-15.7 to -5.7) | **<0.001** | 0.600 |
| Pre-bronchodilator FVC %pred (n=724) | -5.2 (-8.3 to -2.1) | **0.001** | -8.4 (-13.8 to -2.1) | **0.002** | 0.212 |
| Pre-bronchodilator %FEV_1_/FVC (n=745) | 3.1 (1.1 to 5.1) | **0.003** | -7.9 (-11.0 to -4.8) | **<0.001** | **0.013** |
| Asthma control (ACQ) score (n=853) | 0.33 (0.16 to 0.50) | **<0.001** | 1.21 (0.99 to 1.43) | **<0.001** | 0.237 |
| AQLQ score (n=837) | -0.36 (-0.54 to -0.19) | **<0.001** | -1.04 (-0.54 to -0.19) | **<0.001** | 0.137 |
| Activity limitation domain | -0.45 (-0.63 to -0.27) | **<0.001** | -1.00 (-0.63 to -0.27) | **<0.001** | 0.097 |
| Symptoms domain | -0.32 (-0.51 to -0.13) | **0.001** | -1.10 (-1.36 to -0.84) | **<0.001** | 0.264 |
| Emotional function domain | -0.27 (-0.50 to -0.05) | **0.018** | -1.17 (-1.47 to -0.86) | **<0.001** | 0.403 |
| Environmental stimuli domain | -0.30 (-0.51 to -0.09) | **0.005** | -0.79 (-1.07 to -0.51) | **<0.001** | 0.079 |
| No. of exacerbations in previous year (n=455) | 0.4 (-0.1 to 0.8) | **0.122** | 1.0 (0.5 to 1.5) | **<0.001** | 0.848 |
| Airway Inflammation |  |  |  |  |  |
| Sputum %eosinophils (n=116) | -3.2 (-8.3 to 1.8) | 0.205 | 5.5 (-0.1 to 11.1) | 0.055 | 0.386 |
| Sputum %neutrophils (n=115) | 12.1 (1.7 to 22.4) | **0.023** | 4.5 (-9.0 to 17.9) | 0.512 | 0.879 |
| FeNO (ppb) (n=433) | -6.5 (-14.6 to 1.6) | 0.113 | 3.9 (-5.1 to 12.9) | 0.396 | 0.364 |

# ANCOVA models adjusted for asthma severity, obesity, inhaled corticosteroid dose (beclomethasone dipropionate equivalents/day), oral corticosteroid dose (mg/day), biologic use (yes/no) and smoking status (never/ex/current). Definition of abbreviations: ACQ = Asthma Control Questionnaire; AQLQ = Asthma-related Quality of Life Questionnaire; BDP = beclomethasone dipropionate; FeNO = fractional exhaled nitric oxide; FEV_1_ = forced expiratory volume in 1 second; FVC = forced vital capacity; ICS = inhaled corticosteroid; OCS = oral corticosteroid.

**Table S5.** Patient characteristics, according to asthma severity and availability of sputum data.

| **Characteristic** | **Severe asthma** | | | **Non-severe asthma** | | |
| --- | --- | --- | --- | --- | --- | --- |
|  | **Sputum Data Available (n=89)** | **Sputum Data Not Available (n=793)** | **P-value** | **Sputum Data Available (n=28)** | **Sputum Data Not Available (n=109)** | **P-value** |
| BMI (kg/m^2^) (n=1,019) | 31.2 (7.9) | 30.8 (8.1) | 0.731 | 28.0 (6.3) | 28.4 (5.6) | 0.776 |
| Sex (%female) (n=1,017) | 53.9% | 63.6% | 0.074 | 53.6% | 58.7% | 0.623 |
| Age (years) (n=1,000) | 56.4 (14.4) | 54.5 (14.9) | 0.243 | 55.1 (17.2) | 53.8 (16.5) | 0.728 |
| Age at asthma onset (years) (n=881) | 27.5 (21.0) | 22.5 (19.0) | **0.043** | 15.9 (16.2) | 24.6 (20.9) | 0.051 |
| Respiratory Medications |  |  |  |  |  |  |
| ICS dose (BDP µg/day) (n=1,018) | 2000 (1600, 2000) | 1600 (1000, 2000) | **<0.001** | 800 (400, 1300) | 800 (400, 1000) | 0.579 |
| OCS dose for OCS users (mg/day) (n=981) | 10 (5, 13) | 10 (5, 15) | 0.809 | - | 10 (8, 25) |  |
| Pack years of current and ex-smokers (n=372) | 12 (4, 26) | 11 (4, 27) | 0.640 | 7 (1, 31) | 7 (1, 16) | 0.572 |
| Lung function |  |  |  |  |  |  |
| Pre-bronchodilator FEV_1_ %pred (n=765) | 71.8 (22.4) | 64.0 (21.0) | **0.002** | 81.9 (17.8) | 80.0 (20.8) | 0.667 |
| Post-bronchodilator FEV_1_ %pred (n=702) | 76.9 (22.5) | 70.7 (21.8) | **0.025** | 84.7 (16.8) | 82.7 (21.6) | 0.667 |
| Pre-bronchodilator FVC %pred (n=756) | 83.0 (18.4) | 82.0 (19.5) | 0.646 | 88.3 (12.3) | 92.8 (25.5) | 0.397 |
| Pre-bronchodilator %FEV_1_/FVC (n=789) | 66.3 (14.3) | 61.4 (13.6) | **0.003** | 72.5 (12.4) | 68.8 (12.9) | 0.202 |
| Asthma control (ACQ6) score (n=902) | 1.92 (1.11) | 2.40 (1.32) | **0.001** | 0.74 (0.45) | 1.00 (1.07) | 0.201 |
| AQLQ score (n=888) | 5.04 (1.22) | 4.49 (1.37) | **<0.001** | 6.01 (0.69) | 5.80 (1.05) | 0.307 |
| Activity limitation domain | 5.18 (1.25) | 4.58 (1.39) | **<0.001** | 6.04 (0.88) | 5.88 (1.08) | 0.477 |
| Symptoms domain | 4.86 (1.33) | 4.38 (1.46) | **0.004** | 5.96 (0.63) | 5.74 (1.12) | 0.322 |
| Emotional function domain | 5.06 (1.54) | 4.39 (1.70) | **<0.001** | 6.12 (0.87) | 5.78 (1.31) | 0.196 |
| Environmental stimuli domain | 5.15 (1.51) | 4.64 (1.59) | **0.005** | 5.88 (0.98) | 5.68 (1.18) | 0.421 |
| No. of exacerbations in previous year (n=506) | 0 (0, 0) | 1 (0, 3) | **<0.001** | 0 (0, 0) | 0 (0, 0) | 0.150 |
| FeNO (ppb) (n=433) | 13 (6, 22) | 29 (13, 63) | **<0.001** | 19 (12, 25) | 25 (16, 40) | **0.040** |

BMI = body mass index; ICS = inhaled corticosteroid; BDP = beclomethasone dipropionate; OCS = oral corticosteroid; AQLQ = asthma-related quality of life; FeNO = fractional exhaled nitric oxide; - no observations.

**Table S6.** Comorbidities present in patients, according to asthma severity and obesity status.

| **Comorbidity (n, %)** | **Severe Asthma** | | | **Non-severe Asthma** | | | **P-value*** |
| --- | --- | --- | --- | --- | --- | --- | --- |
|  | **BMI <30kg/m^2^**  **(n=459)** | **BMI ≥30 kg/m^2^**  **(n=423)** | **P-value** | **BMI <30 kg/m^2^**  **(n=97)** | **BMI ≥30 kg/m^2^**  **(n=40)** | **P-value** |  |
| **Upper airways and Pulmonary** |  |  |  |  |  |  |  |
| Allergic rhinitis | 228 (49.7%) | 209 (49.4%) | 0.938 | 50 (51.6%) | 18 (45.0%) | 0.486 | 0.921 |
| Chronic rhinosinusitis | 39 (8.5%) | 39 (9.2%) | 0.706 | 5 (5.2%) | 3 (7.5%) | 0.595 | 0.629 |
| Nasal polyps | 88 (19.2%) | 81 (19.2%) | 0.993 | 7 (7.2%) | 7 (17.5%) | 0.071 | **0.037** |
| Vocal cord dysfunction | 24 (5.2%) | 34 (8.0%) | 0.093 | 1 (1.0%) | 3 (7.5%) | **0.041** | **0.048** |
| Obstructive sleep apnoea | 33 (7.2%) | 106 (25.1%) | **<0.001** | 4 (4.1%) | 6 (15.0%) | **0.026** | **<0.001** |
| Bronchiectasis | 39 (8.5%) | 29 (6.9%) | 0.361 | 3 (3.1%) | 1 (2.5%) | 0.851 | 0.167 |
| Chronic obstructive pulmonary disease | 47 (10.2%) | 36 (8.5%) | 0.380 | 1 (1.0%) | 4 (10.0%) | **0.011** | **0.033** |
| Hyperventilation dysfunctional breathing | 9 (2.0%) | 11 (2.6%) | 0.524 | 1 (1.0%) | 0 (0.0%) | 0.519 | 0.576 |
| Allergic bronchopulmonary aspergillosis | 32 (7.0%) | 10 (2.4%) | **0.001** | 2 (2.1%) | 1 (2.5%) | 0.873 | **0.005** |
| **Extrapulmonary** |  |  |  |  |  |  |  |
| Metabolic disease |  |  |  |  |  |  |  |
| Diabetes | 28 (6.1%) | 80 (18.9%) | **<0.001** | 4 (4.1%) | 7 (17.5%) | **0.009** | **<0.001** |
| Cardiovascular disease |  |  |  |  |  |  |  |
| Heart attack | 10 (2.2%) | 10 (2.4%) | 0.853 | 2 (2.1%) | 1 (2.5%) | 0.873 | 0.996 |
| Stroke | 1 (0.2%) | 5 (1.2%) | 0.082 | 0 (0.0%) | 2 (5.0%) | **0.027** | **0.005** |
| Other cardiovascular disease | 75 (16.3%) | 91 (21.5%) | 0.050 | 22 (22.7%) | 19 (47.5%) | **0.004** | **<0.001** |
| Psychiatric disease |  |  |  |  |  |  |  |
| Anxiety | 63 (13.7%) | 91 (21.5%) | **0.002** | 14 (14.4%) | 5 (12.5%) | 0.766 | **0.014** |
| Depression | 73 (15.9%) | 104 (24.6%) | **0.001** | 16 (16.5%) | 6 (15.0%) | 0.828 | **0.008** |
| Gastro-oesophageal reflux disease | 173 (37.7%) | 203 (48.0%) | **0.002** | 24 (24.7%) | 21 (52.5%) | **0.002** | **<0.001** |
| Osteoporosis | 63 (13.7%) | 55 (13.0%) | 0.753 | 7 (7.2%) | 4 (10.0%) | 0.586 | 0.338 |
| Allergic disease |  |  |  |  |  |  |  |
| Eczema | 67 (14.6%) | 79 (18.7%) | 0.103 | 16 (16.5%) | 10 (25.0%) | 0.248 | 0.206 |
| Anaphylaxis | 26 (5.7%) | 35 (8.3%) | 0.127 | 5 (5.2%) | 3 (7.5%) | 0.595 | 0.417 |

**Figure S1.** Proportion of patients who had anxiety with a) severe asthma, and b) non-severe asthma, according to obesity status. And proportion of patients who had depression with c) severe asthma, and d) non-severe asthma, according to obesity status.

**Figure S2.** Change in a) FEV_1_, b) FVC, c) asthma control (ACQ6), d) asthma-related quality of life (AQLQ score), e) daily oral corticosteroid dose, and f) number of asthma exacerbations experienced in the preceding 12 months, in patients with severe asthma prescribed biologic therapy according to obesity status. P-values represent between group differences in change, while significant within group changes are also indicated.
